# Effect of Chinese herbal compounds on ocular fundus signs and vision in conventional treated-persons with non-proliferative diabetic retinopathy: A systematic review and meta-analysis

**DOI:** 10.3389/fendo.2022.977971

**Published:** 2022-08-11

**Authors:** Xiaodong Li, Jiaqi Zhang, Runxi He, Xiaojuan Su, Zhilin Li, Xuejun Xie

**Affiliations:** ^1^ Eye School of Chengdu University of Traditional Chinese Medicine, Chengdu, China; ^2^ Ophthalmology, Affiliated Hospital of Chengdu University of Traditional Chinese Medicine, Chengdu, China

**Keywords:** Chinese medicine compounds, diabetic retinopathy, Chinese and Western medicine combination, safety evaluation, meta-analysis (as topic)

## Abstract

**Background:**

Changes in fundus signs and loss of visual acuity are an important basis for screening and treating diabetic patients with retinopathy, and conventional Western medicine is moderately effective in treating diabetic retinopathy(DR),To systematically evaluate the effectiveness and safety of Chinese herbal compounds(CHCs) in the combined treatment of diabetic retinopathy.

**Method:**

Six electronic databases, including PubMed, were searched to screen eligible literature. Randomized controlled trials of non-proliferative diabetic retinopathy(NPDR) were included, in which the control group was treated with conventional Western-based drugs or retinal laser photocoagulation, and the intervention group was treated with CHCs in combination based on the control group.The Cochrane Risk of Bias Assessment Tool was used to evaluate the quality of the literature, and the RevMan 5.4 software was used for statistical analysis.

**Results:**

Compared with Conventional group alone,CHCs group was superior at improving clinical efficacy [RR=1.29, 95%CI=(1.23, 1.36),P<0.01] and best corrected visual acuity(BCVA) [MD=0.10,95%CI=(0.09,0.12),P<0.01],it was also superior at reducing the number of microangiomas [MD=-2.37, 95%CI=(-3.26, -1.49),P<0.01], microangioma volume [MD=-4.72, 95%CI=(-5.14, -4.29), P<0.01], hemorrhagic spots [MD=-2.05, 95%CI=(-2.51,-1.59), P<0.01], hemorrhagic area [MD=-0.76, 95%CI=(-1.06, -0.47), P<0.01], hard exudates [MD= -1.86, 95%CI=(-2.43, -1.28), P<0.01], cotton lint spots [MD= -0.93, 95%CI= (-1.31, -0.55), P<0.01], central macular thickness(CMT) [SMD=-1.52, 95%CI=(-1.85, -1.19),P<0.01], Chinese medicine evidence score [SMD=-1.33,95%CI=(-1.58, -1.08),P<0.01], fasting blood glucose (FBG) [SMD=-0.47, 95%CI=(-0.61,-0.33),P<0.01], 2h postprandial blood glucose(2hPBG) [SMD=-0.87, 95% CI=(-1.06, -0.67), P<0.01], glycosylated hemoglobin (HbAlc) [SMD=-0.76, 95%CI=(-1.16, -0.3),P<0.01], total cholesterol(TC) [SMD=-0.33,95%CI=(-0.51,-0.16),P<0.01],and CHCs group with less adverse events occurred [RR=0.46, 95%CI=(0.29, 0.74),P<0.01].

**Conclusion:**

CHCs combined with conventional medicine for NPDR has better clinical efficacy and higher safety, but the above findings need further validation in more large sample, multicenter, and low-bias RCTs due to the limitation of the quality and quantity of included literature.

**Systematic Review Registration:**

https://www.crd.york.ac.uk/prospero/, identifier CRD42022342137.

## Highlights

1. Combination therapy with CHCs can improve clinical effective rate and BCVA for NPDR patients in this review.
2. CHCs as an add-on therapy showed clinically and statistically significant reductions in microangioma, hemorrhage, central macular thickness, FBG, 2hPBG, HbAlc, TC, and Chinese medicine evidence score for NPDR.
3. The outcomes can evaluate the therapeutic effects of CHCs in the treatment of NPDR objectively, indicating that CHCs might be used as a complementary and alternative approach to prevent, delay and reverse NPDR progression.

## Introduction

According to the 10th edition of the IDF Diabetes Atlas the global prevalence of diabetes in people aged 20-79 years is estimated at 10.5% in 2021 and is expected to rise to 12.2% (783.2 million people) by 2045 (1) with an inevitable subsequent increase in the prevalence of DR. China has the largest diabetes population in the world, and a meta-analysis of the epidemiology of DR in China showed that the combined prevalence of DR, NPDR and PDR among diabetic patients was 18.45%, 15.06% and 0.99%, respectively, and the prevalence of DR peaked between 60 ~ 69 years of age and increased sharply with the duration of diabetes, in addition to insulin In addition, insulin therapy, elevated FBG levels, and elevated glycated hemoglobin concentrations have been shown to be associated with a higher prevalence of DR in diabetic patients (2) DR is a common complication of diabetes that leads to lesions in the retinal neurovascular unit, and is the top-ranked fundus retinal disease in terms of risk of blindness (3). DR is a common complication of diabetes mellitus that leads to retinal neurovascular unit lesions and is the number one risk of blindness.

NPDR is characterized by retinal hemangiomas, hemorrhages, hard exudates, cotton wool spots and IRMA signs, while PDR is characterized by retinal neovascularization, vitreous or preretinal hemorrhages (4). How to prevent and control early DR as well as to control the progression of NPDR to PDR and to maximize the protection and recovery of vision in patients with DR is a challenge and a hot topic of research for scholars. At present, the conventional therapies to prevent and control the progression of DR include glucose lowering, microcirculation improvement, nerve nutrition, retinal laser photocoagulation, vitreous cavity injection of anti-vascular endothelial growth factor and vitrectomy surgery (5). The efficacy of TCM treatments in preventing and treating DR, especially NPDR progression, has been demonstrated in a growing number of studies that CHCs and TCM preparations as adjunctive therapies can improve the effectiveness of conventional treatment of DR (6). A recent meta-analysis showed that, compared with conventional treatment of NPDR in Western medicine, the combination of Chinese and Western treatments supplemented with proprietary Chinese medicines improved retinal microangiomas, hemorrhages, CMT, BCVA, FBG, and HbAlc was improved. In the Chinese Physicians Association’s 2021 Guidelines for the Integrated Treatment of Diabetic Retinopathy (7), However, there are no systematic data on the evaluation of fundus signs, visual acuity, and safety of CHCs combined with conventional Western medicine in the treatment of NPDR. Therefore, this study was conducted to evaluate the fundus signs, visual acuity, and safety of CHCs as an adjunctive therapy against NPDR in order to provide high-quality evidence-based evidence to help clinicians choose a better treatment strategy.

## Methods

### Search strategy

**Figure 1:**
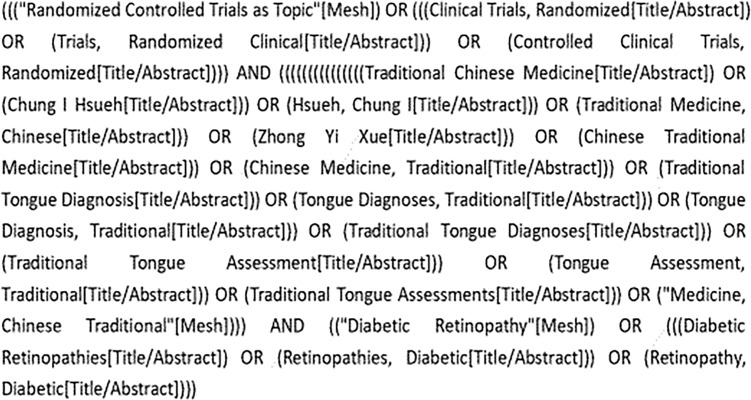
PubMed literature search strategy.

The RCTs of 15 CHCs (specific prescriptions for specific diseases) for DR recommended in the Guidelines for the Integrated Treatment of Diabetic Retinopathy were searched in CNKI, Wanfang, VIP, PubMed, The Cochrane Library, and Embase databases, and the search period was from the database to June 2022. The Chinese search terms included “diabetic retinopathy”, “thirsty eye disease”, “Chinese medicine”, “randomized controlled clinical study”, and “da ming drink. “The English search terms included “Diabetic Retinopathy”, “Chinese herbal medicine” and “Chinese herbal medicine”. “Chinese herbal medicine”, “Medicine, Chinese Traditional The English search terms included “Diabetic Retinopathy”, “Chinese herbal medicine”, “Randomized controlled trials”, etc. The search strategy was based on PubMed, for example, as shown in **Figure 1**.

### Study population

For patients who meet the diagnosis of DR, the diagnostic criteria refer to the Multidisciplinary Chinese Expert Consensus on the Prevention and Treatment of Diabetes-Related Eye Diseases (2021 Edition) developed by the Retinopathy Group of the Diabetes Branch of the Chinese Medical Association (8).

### Study selection

#### The inclusion criteria were as follows

(1) Type of study: clinical RCTs in Chinese and English; (2) The subjects included in the study were NPDR patients with confirmed type 2 diabetes mellitus, regardless of gender, age and duration of disease; (3) Interventions: the control group was conventional Western medical therapy, including blood glucose control, blood pressure lowering, lipid regulation, microcirculation improvement, retinal laser photocoagulation and vitreous cavity injection of anti-vascular endothelial factor drugs, etc. (4) Outcome indicators: at least one of the following should be included: (1) effectiveness evaluation indicators: total clinical efficiency, best corrected visual acuity (BCVA), fundus signs including microangiomas, hard exudates, cotton wool spots, hemorrhages and CMT, etc. (2) Indicators related to primary diabetes: FBG, 2h-PBG, HbA1c; (3) Lipid indicators: serum TC and triglyceride (TG); (4) Inflammatory factors: tumor necrosis factor-α (TNF-α), etc.; (5) Other indicators: Chinese medicine evidence evaluation score; (6) Safety evaluation indicators: adverse reactions (5) General data between the two groups. (5) The differences in general data between the two groups were not statistically significant, well-balanced and comparable.

#### The exclusion criteria were as follows

(1) literature with duplicate or incomplete data publication, inappropriate statistical methods, and less rigorous design; (2) literature with only abstracts that do not allow access to the full text; (3) literature from animal experiments, reviews, case reports, retrospective studies, and other non-randomized controlled clinical research categories; (4) literature with patients with PDR in the study population, or patients with a combination of other serious retinal diseases such as retinal vein obstruction, uveitis, or a combination of systemic severe organic pathologies such as cardiac, cerebral, hepatic, and renal diseases; (5) the control group used TCM-type therapeutic interventions and the treatment group did not incorporate Western medical treatment protocols; (6) the interventions in the treatment group included other TCM external therapies, such as acupuncture, moxibustion, and tui na.

### Data extraction and quality assessment

Literature screening was completed independently by the 2 groups of researchers, and after excluding studies that clearly did not meet the inclusion criteria, the abstracts and full texts were further read to determine whether the inclusion criteria were met. Information from the included literature was extracted and cross-checked. In case of disagreement, confer and reach consensus with the 3rd investigator. Information extracted included: 1. basic information on RCTs, baseline status of patients in the trial and control groups; 2. interventions, outcome indicators, and missed visits and treatment; 3. indicators of study quality including whether the randomization method was correct, whether allocation concealment was achieved, whether blinding was used, whether there were missed visits and withdrawals, whether there was selective reporting bias, and whether there were other biases.

### Risk of bias evaluation

Methodological quality was evaluated using the quality assessment criteria for randomized controlled trials recommended in the Cochrane Systematic Evaluation Manual version 5.1.0: method of random allocation; whether allocation was concealed in real time; whether the study subjects, treatment regimen implementers, and study outcome measures were blinded; whether data were complete; whether there was selective reporting of results; and whether there were other biases, including early trial stoppage, baseline un balance, etc. A “yes” (low risk of bias), “no” (high risk of bias), or “unclear” (lack of relevant information or uncertainty about bias) was used. The methodological quality was evaluated independently by two evaluators, with third-party input and agreement in case of disagreement. When more than 10 studies were included in the analysis, funnel plots were drawn to analyze the presence of publication bias.

### Statistical analysis

Meta-analysis was performed using the Rev Man 5.4 software provided by the Cochrane Collaboration Network. The mean difference (mean difference, MD) or standard mean difference SMD and its 95% CI were used to express the measurement data, and the relative risk (RR) and its 95% CI were used to express the count data. Studies were tested for heterogeneity, and if there was no heterogeneity or small heterogeneity (I^2^ ≤50%,P≥0.05), a fixed-effects model was used to calculate the combined effect size; conversely, if the heterogeneity was large (I^2^ > 50%, P< 0.05), a random-effects model was used to combine the effect sizes. p<0.01 indicated a statistically significant difference.

## Results

### Literature search results and basic characteristics

**Figure 2:**
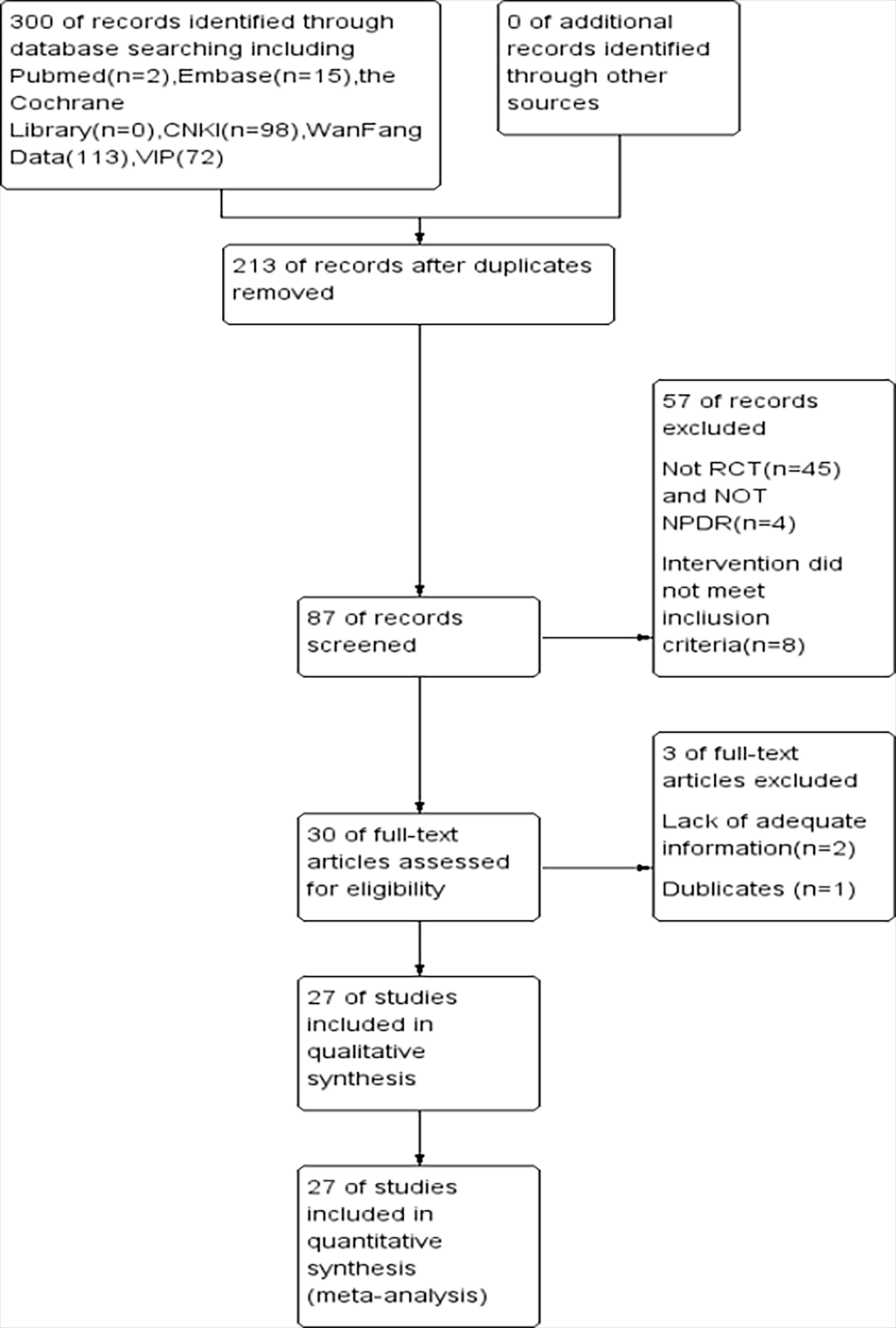
Flow chart of literature screening.

After searching the relevant databases, a total of 98 literature were searched, and 27 RCTs were finally screened by nadir criteria (9–35), including 2144 patients with DR, the basic information of the literature is shown in **Table 1**, and the screening flow chart is shown in **Figure 2**.

**Table 1:** Basic characteristics of included RCTs.

Study ID	Sample sizeC/T		ageC/T	Course of DMC/T	Random method	control group/C	Intervention group/T	Intervention time(month)	Mainindicators	Adverse reactions C/T
Zhang Lianhuan 2021 (9)	80/80		59.72 ± 13.16 /59.63 ± 13.08	4.17 ± 1.48 /4.21 ± 1.52	Random number table method	CW	CW+Bushen Huoxue Mingmu Tang	1	A/C2/E/F/H/I	9/2
Li Xiaozhong 2017 (10)	38/37		53.69 ± 7.52/ 53.97 ± 7.38	**/**	Random number table method	CW	CW+Ziyin Mingmu Decoction	3	B/C2	**/**
Luo Dan 2011 (11)	32/32		62.3 ± 3.7/61.6 ± 3.1	6.8 ± 1.8/ 7.1 ± 1.6	simple random method	CW	CW+Ziyin Mingmu Decoction	3	B/C2/E	**/**
Luo Xiaoqin 2017 (12)	23/23		60.4 ± 8.6/60.6 ± 8.7	7.5 ± 3.4/ 7.4 ± 3.2	simple random method	CW	CW+Ziyin Mingmu Decoction	3	B/C2	**/**
Tan Hui 2015 (13)	47/50		60.4 ± 10.2 /61.3 ± 11.4	8.8 ± 4.9 /8.6 ± 5.4	Random number table method	CW	CW+ Bushen Huoxue Mingmu Tang	3	A/C2/D/G	**/**
Xiao Haijing 2020 (14)	23/29		54.81 ± 8.44/52.68 ± 8.91	**/**	simple random method	CW	CW+Ziyin Mingmu Decoction	3	A/B	**/**
Gao Wei 2017 (15)	40/40		47.7 ± 8.1/46.9 ± 7.5	**/**	simple random method	CW	CW+Yiqi Yangyin Experience Formula	3	I	**/**
Zhang Shifen 2017 (16)	38/36		**/**	**/**	simple random method	CW	CW+ Xiaoke Mingmu Decoction	3	C2/D/F/G	**/**
Ye Yingyin 2019 (17)	54/54		67.18 ± 14.21/ 62.85 ± 11.62	8.22 ± 5.02/ 7.31 ± 4.79	simple random method	CW	CW+ Xiaoke Mingmu Decoction	3	C2/E	17/7
Zhang Yuxian 2020 (18)	73/79		51.35 ± 10.12	6.22 ± 5.84	simple random method	CW	CW+Da Ming Yin	3	A/C2	3/2
Wu Hongya 2019 (19)	48/48		50.5 ± 9.2/ 51.9 ± 9.5	**/**	Random number table method	CW	CW+Tangwang Mingmu Decoction	1	A/C2/E/F	**/**
Liu Fengtong 2018 (20)	33/33		45.57 ± 1.89/ 45.87 ± 1.76	4.06 ± 1.36/ 4.18 ± 1.52	simple random method	CW	CW+Tangwang Mingmu Decoction	2	A/C2/E/F	1/1
Shi Wei 2019 (21)	100/100		51.6 ± 9.2/ 54.6 ± 8.5	9.8 ± 2.4/ 10.2 ± 2.6	Random number table method	CW	CW+Shenqing Jiangzhuo Tongluo Mingmu pre-scription	2	B/E/G	**/**
Wang Yu 2020 (22)	40/40		54.03 ± 5.59 /55.83 ± 5.56	2.55 ± 0.70/ 2.20 ± 0.90	simple random method	CW	CW+Tangwang Mingmu Decoction	4	A/C2/E/F	**/**
Xia Wei 2016 (23)	34/37		49.75 ± 8.14/ 57.50 ± 4.25	13.28 ± 2.14/ 14.19 ± 1.7	simple random method	CW	CW+Mimenhuang Formula	2	C2/H	**/**
Chen Yanglei 2021 (24)	42/42		56.14 ± 3.22/ 56.32 ± 2.24	12.14 ± 3.25/ 12.24 ± 3.12	Random number table method	CW	CW+Mimenhuang Formula	3	C2	**/**
Hu Xiaodan 2017 (25)		40/40	52.31 ± 3.07/ 52.35 ± 3.11	11.73 ± 5.03/ 11.62 ± 4.20	simple random method	CW	CW+Mimenhuang Formula	3	B/C2	5/4
Yan Jing 2010 (26)		32/31	**/**	**/**	simple random method	CW	CW+Mimenhuang Formula	3	B/D/E/G	**/**
Zhang Yuanzhong2017 (27)		30/30	61.47 ± 12.53/ 59.65 ± 13.20	12.53 ± 3.68/ 11.21 ± 4.56	simple random method	CW	CW+Huayu Mingmu mixture	3	B/E/F/G	**/**
Sun Xiaoyan 2016 (28)		16/19	62.50 ± 11.5/ 60.89 ± 13.2	11.53 ± 3.71/ 12.21 ± 4.76	simple random method	CW	CW+Huayu Mingmu mixture	3	C2/D/E/F/G	**/**
Luo Xianling 2022 (29)		46/46	60.53 ± 13.06/59.52± 12.87	60.53 ± 13.06/ 12.74± 2.70	Random number table method	CW	CW+Huayu Mingmu mixture	1	A/B/C2/E/F/H	5/4
Zhang Yinjian 2012 (30)		34/35	66.12 ± 7.89/ 62.37 ± 9.73	13.88 ± 9.47/ 13.46 ± 8.93	simple random method	CW	CW+Heying Prescription	3	A/C2	**/**
Zhou Jing 2022 (31)		30/30	50.56 ± 5.12/ 50.77 ± 5.09	10.23 ± 2.36/ 10.45 ± 2.28	simple random method	CW	CW+Danhuang Mingmu Decoction	4	A/H/I	7/2
Hu Qi 2022 (32)		20/19	57.4 ± 4.09/ 56.95 ± 10.26	9.63 ± 3.03/ 9.54 ± 3.05	Random number table method	CW	CW+Danhuang Ming mu Decoction	3	A/C2	1/0
Song Flame 2021 (33)		21/22	56.82 ± 8.95/ 59.05 ± 7.51	10.36 ± 4.86/ 11.05 ± 4.86	simple random method	CW	CW+Danhuang Mingmu Decoction	3	A/C2/D/H	**/**
Xia Xiangjun 2012 (34)		18/18	**/**	**/**	Random number table method	CW	CW+Da Ming Yin	1	A/C2	**/**
Liu Jin 2019 (35)		29/33	48.21 ± 4.19	15.11 ± 9.64	simple random method	CW	CW+Mimenhuang Formula	4	C2	**/**

T, Intervention group; C, control group; CW, conventional treatment of Western medicine; A, BCVA; B, Ocular Fundus Signs (microhemangioma,hemorrhages, hard exudates and cotton lint spots); C2, total clinical response rate; D, TCM syndrome evaluation integral; E, FBG and 2hPBG; F, HbA1c; G, TC and TG; H, CMT; I,TNF-α; /, no records.

### Results of the risk of bias evaluation

**Figure 3:**
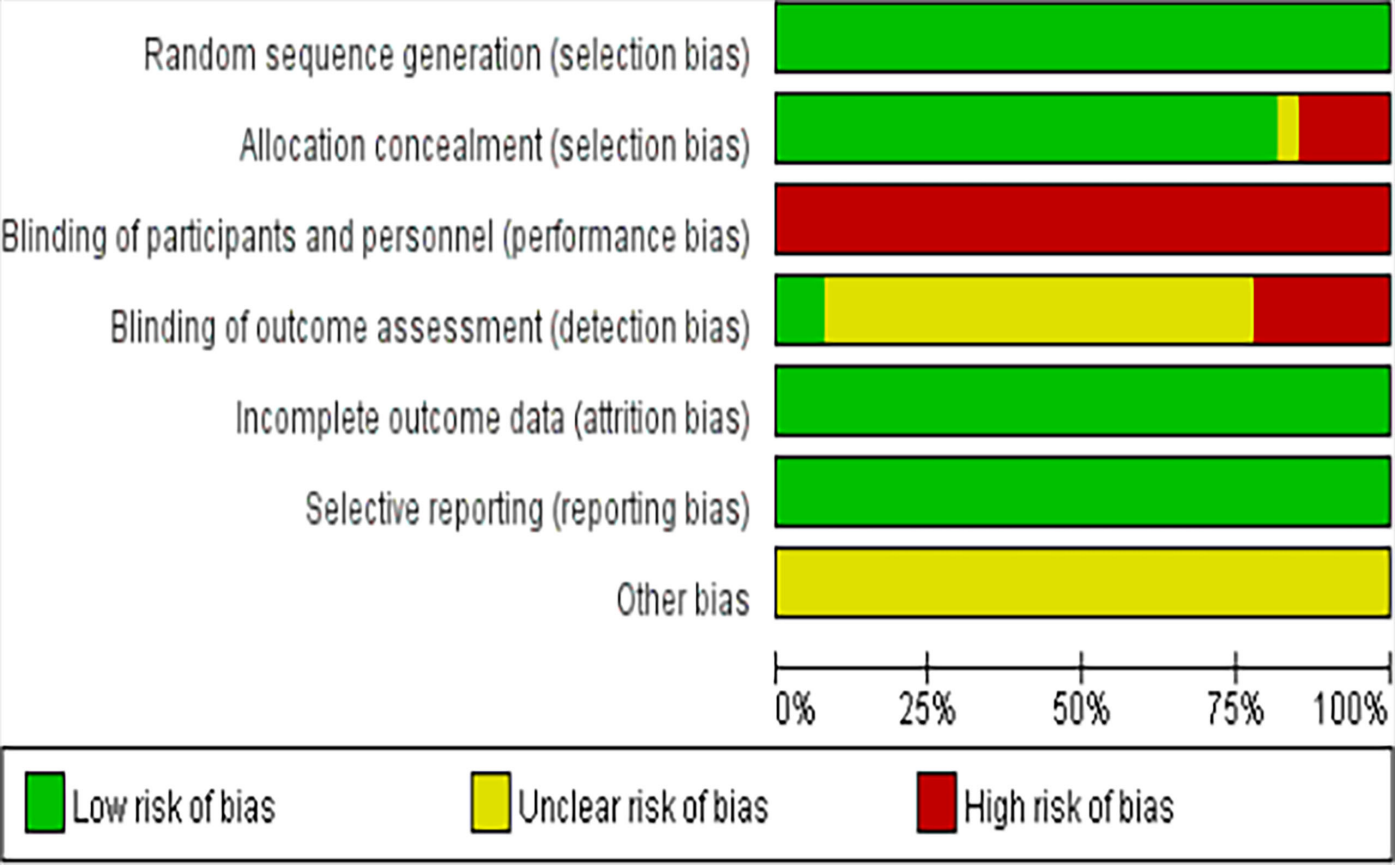
Risk of bias assessment graph for included RCTs.

**Figure 4:**
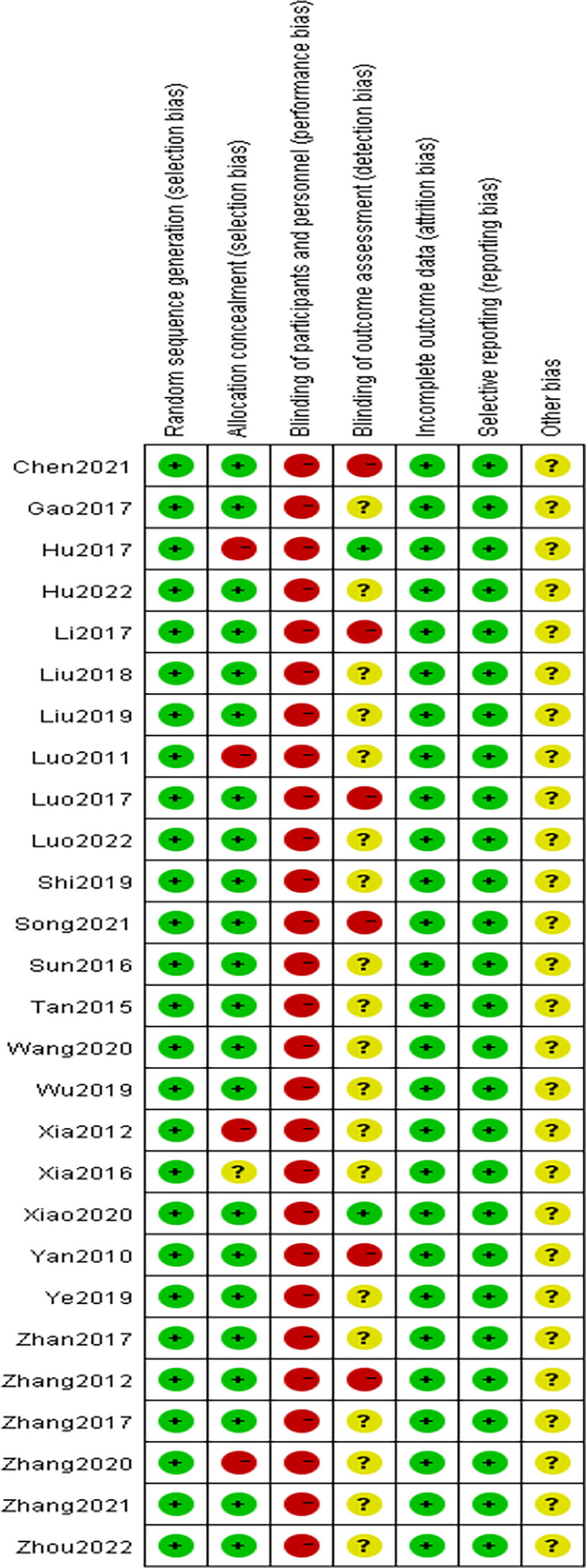
Distribution of risk of bias of included RCTs.

The methodological quality of the 27 included studies (9–35) was evaluated using the quality evaluation criteria for randomized controlled trials recommended by the Cochrane Systematic Evaluation Manual, version 5. 1. 0, and the results are shown in **Figures 3** and **4**.

### Total clinical effectiveness

**Figure 5:**
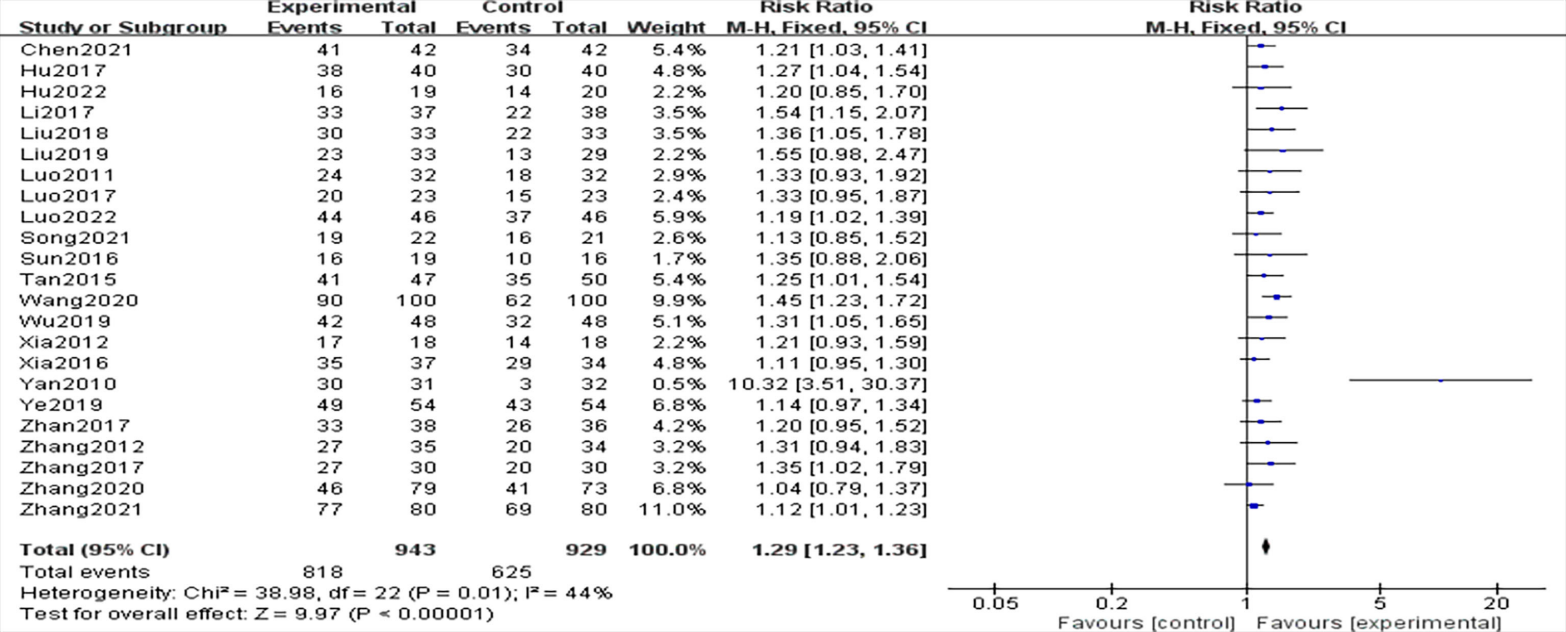
Forest plot of total efficiency rate comparison.

A total of 23 RCTs (9–13, 16–20, 22–25, 28–30, 32–35) provided data on the total clinical efficiency of the studies. Heterogeneity among the included studies was low (P=0.01,I^2 =^ 44%), so using fixed-effects model analysis, the total clinical effective rate was significantly higher in the CHCs group than in the control group, and the difference was statistically significant [n=1872, RR=1.29, 95%CI (1.23,1.36),P<0.01], indicating that CHCs combination therapy improves the total clinical effective rate, as shown in **Figure 5**.

### Best corrected visual acuity

**Figure 6:**
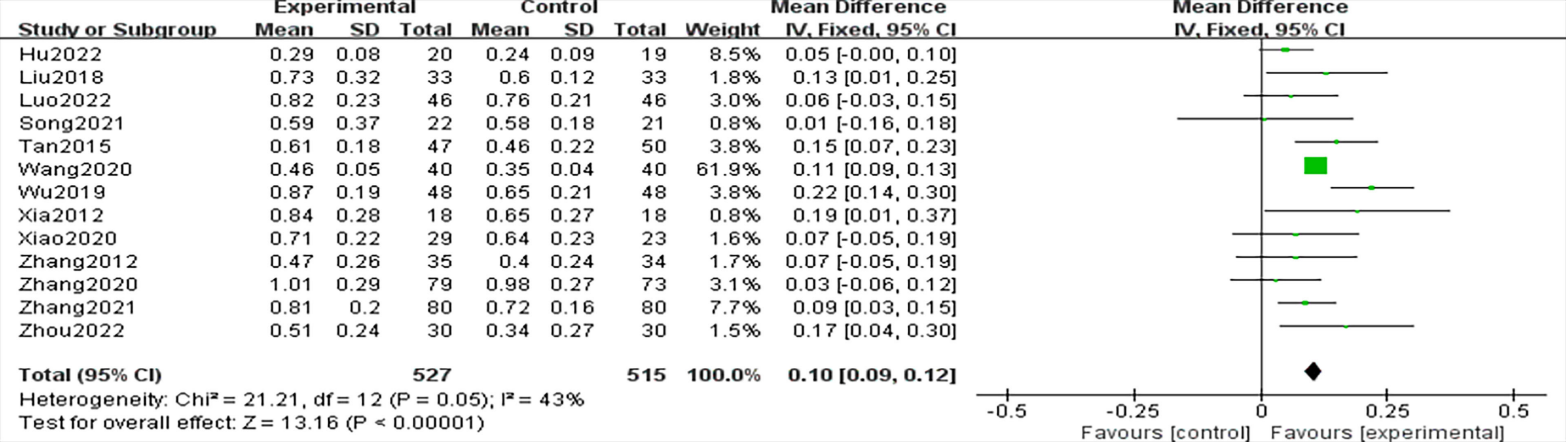
Forest plot of BCVA.

A total of 13 RCTs (9, 13, 14, 18–20, 22, 29–34) reported BCVA data. Heterogeneity among the included studies was not significant, (P=0.05,I^2 =^ 43%), and analysis using a fixed effects model showed that the BCVA’s in the CHCs group after treatment were significantly higher than those in the control group, with a statistically significant difference [n=1042, MD=0.10, 95% CI (0.09,0.12), P<0.01], indicating that the CHCs combination therapy facilitated the improvement of BCVA, as shown in **Figure 6**.

### Microangiomas volume and counts

A total of 5 RCTs (10–12, 14, 25) were conducted on the counts of microangiomas in 317 patients with DR, and meta-analysis showed that the counts of microangiomas in the CHCs group were significantly less than those in the control group, indicating that CHCs combination therapy could significantly reduce the number of microangiomas;2 RCTs (21, 29) reported on the area of microangiomas in 292 patients with DR, and meta-analysis showed that the CHCs group was significantly more effective in reducing the area of microangiomas in efficacy than the control group, indicating that CHCs combination therapy significantly reduced the area of microangiomas, as shown in **Table 2**.

**Table 2:** Meta-analysis results of microhemangioma.

Ending indicators	Number of studies included	Meta-analysis results		effect model	Heterogeneity test results	
		MD(95% CI) P value			P-value	I^2^ (%)
Microangiomas counts	5	-1.26 (-1.51,-1.02)	<0.01	fixation	0.28	21
Microangioma Volume	2	-4.72 (-5.14,-4.29)	<0.01	fixation	0.60	0

### Hemorrhage

2 RCTs (11, 12) reported bleeding point counts in 110 patients with DR, and meta-analysis showed that the CHCs group had better efficacy than the control group in reducing hemorrhage, indicating that CHCs combination therapy could significantly reduce the hemorrhage; 3 RCTs (10, 14, 29) counted bleeding area data in 219 patients with DR, and meta-analysis showed that the efficacy of the CHCs group in reducing hemorrhage area remained superior to the control group, indicating that CHCs combination therapy could significantly reduce the hemorrhage Area, as shown in **Table 3**. There were too few included literatures in this group to conduct subgroup analysis. No source of heterogeneity was found after exclusion of included literatures one by one, which was considered to be caused by clinical heterogeneity or small sample size.

**Table 3:** Meta-analysis results of hemorrhagic.

Ending indicators	Number of studies included	Meta-analysis results			Heterogeneity test results	
		MD(95% CI) P value		effect model	P-value	I^2^ (%)
Hemorrhage	2	-2.05 (-2.51,1.59)	<0.01	fixation	0.83	0
Hemorrhage Area	3	-0.76 (-1.06,-0.47)	<0.01	randomly	<0.01	94

### Hard exudates, cotton lint spots and CMT

Two studies (11, 12) reported hard exudate and lint spots in 110 DR patients, and meta-analysis showed that the number of hard exudate and lint spots in the CHCs group was lower than that in the control group, indicating that CHCs combination therapy could significantly reduce hard exudate and cotton lint spots; a total of 5 RCTs (9, 23, 29, 31, 33)counted CMT data in 426 DR patients, and meta-analysis showed that CMT in the CHCs group was significantly lower than that in the control group, indicating that CHCs combination therapy significantly reduced CMT, as shown in **Table 4**.

**Table 4:** Hard exudation, cotton lint spot and CMT meta-analysis results.

Ending indicators	Number of studies included	Meta-analysis results			Heterogeneity test results	
		MD(95% CI) P value		effect model	P-value	I^2^ (%)
hard exudates	2	-1.86 (-2.43,1.28)	<0.01	fixation	0.87	0
cotton lint spots	2	-0.93 (-1.31,-0.55)	<0.01	fixation	0.44	0
CMT	5	-1.52 (-1.85,-1.19)	<0.01	randomly	0.07	53

### Chinese medicine evidence score

**Figure 7:**
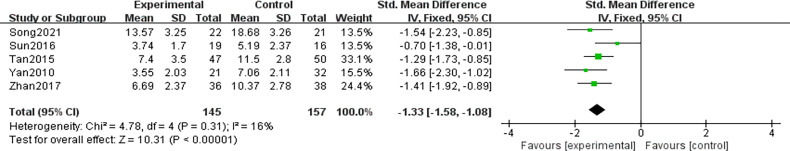
Forest plot of TCM syndrome integral comparison.

A total of 5 RCTs (13, 16, 26, 28, 33) were conducted on the TCM evidence score data of 302 DR patients, and there was no significant heterogeneity among the included studies (P=0.31,I^2 =^ 16%), and the TCM evidence efficacy score after treatment in the CHCs group was significantly lower than that in the control group using fixed-effects model analysis, and the difference was statistically significant [SMD=-1.33, 95% CI (-1.58, -1.08),P<0.01], indicating that the CHCs combination treatment could effectively reduce the TCM evidence score, as shown in **Figure 7**.

### Blood glucose, blood lipids

A total of 9 RCTs (9, 11, 17, 20, 21, 26–29) reported FBG in 848 patients with DR data, and meta-analysis showed that FBG was significantly lower in the CHCs group than in the control group, indicating that CHCs combination therapy could significantly reduce FBG levels; 5 RCTs (9, 17, 20–22) recorded data on 2hFPG in 614 DR patients, and meta-analysis showed that 2hFPG was significantly lower in the CHCs group than in the control group, indicating that CHCs combination therapy could significantly reduce 2hFPG levels; in addition, 8 RCTs (9, 16, 19, 20, 22, 27–29)reported data on HbA1c in 663 DR patients, and meta-analysis showed that HbA1c in the treatment group was lower than that in the control group, indicating that CHCs combination therapy could significantly reduce HbA1c levels. The source of heterogeneity in this group was considered to be clinical heterogeneity or the small sample size.

A total of 6 RCTs (13, 16, 21, 26–28)reported data on TC in 529 DR patients, and meta-analysis showed that TC in the CHCs group was significantly lower than that in the control group, indicating that CHCs combination therapy could significantly reduce TC levels; A total of 5 RCTs (13, 16, 26–28) reported data on TG in 392 DR patients, and meta-analysis showed that TG in the CHCs group was slightly higher than that in the control group, indicating that CHCs combination therapy was comparable to conventional Western treatment of TG, all of which are shown in **Table 5**.

**Table 5:** Meta-analysis results of blood glucose and blood lipid.

Closing indicators	Number of studies included	Meta-analysis results			Heterogeneity test results	
		SMD(95%CI) P-value		effect model	P-value	I^2^ (%)
FBG	9	-0.47 (-0.61,-0.33)	<0.01	fixation	0.08	43
2hPBG	5	-0.87 (-1.06, -0.67)	<0.01	fixation	0.26	25
HbA1c	8	-0.76 (-1.16, -0.3)	<0.01	randomly	<0.01	83
TC	6	-0.33 (-0.51,-0.16)	<0.01	fixation	0.15	38
TG	5	0.11 (-0.29,0.30)	0.97	fixation	0.10	48

### Inflammatory factors

**Figure 8:**
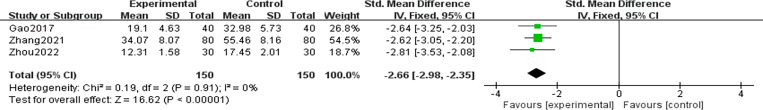
Forest plot of TNF-α comparison.

A total of 3 RCTs (9, 15, 31) reported data on TNF-α in 300 patients with DR, with no significant heterogeneity among the included studies (P=0.91,I^2 =^ 0%), and a statistically significant difference in TNF-α after treatment in the CHCs group compared to the control group using a fixed effects model [SMD=-2.66, 95%CI (-2.98,-2.35) P<0.01], indicating that CHCs combination therapy significantly reduced TNF-α levels, as shown in **Figure 8**.

### Adverse reactions

**Figure 9:**
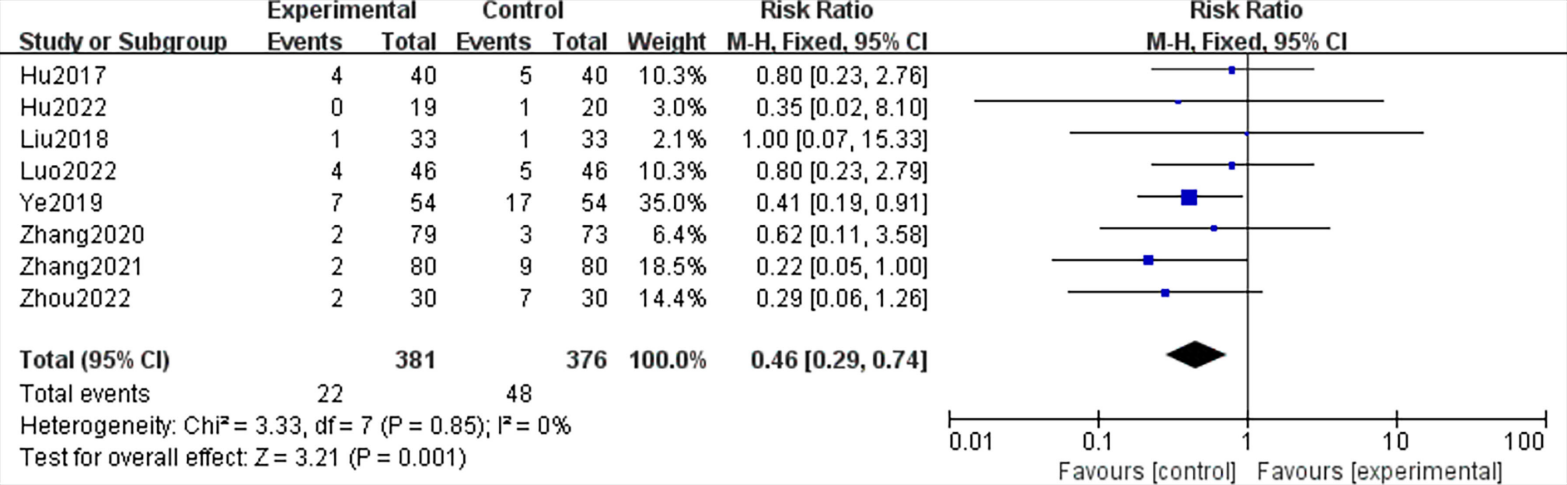
Forest plot of comparative adverse reactions.

A total of 8 RCTs (9, 17, 18, 20, 25, 29, 31, 32) reported data on 757 patients with DR who developed adverse reactions, with no significant heterogeneity among the included studies (P=0.85,I^2 =^ 0%), and the risk of adverse reactions was significantly lower in the CHCs group than in the control group using a fixed effects model, with a statistically significant difference [RR=0.46, 95% CI(0.29,0.74),P<0.01], indicating that the safety of CHCs combination therapy was good, as shown in **Figure 9**.

### Publication bias

**Figure 10:**
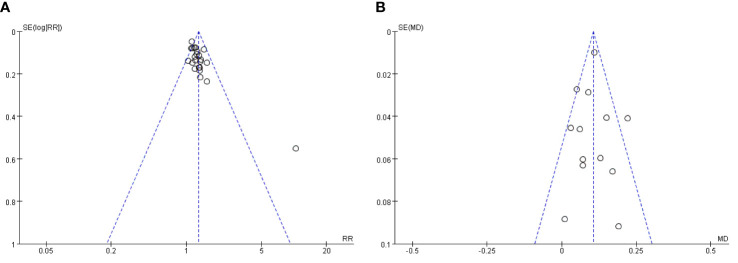
**(A)** Publication bias funnel plot of total clinical response rate. **(B)** Published bias funnel plot of BCVA

Funnel plots were plotted for studies with >10 publications for the combined outcome indicators, and the total clinical effectiveness after treatment showed a significantly asymmetric funnel plot (**Figure 10A**), indicating publication bias in the included studies, which may be due to the small number of included publications and difficulty in including negative outcomes; the BCVA showed a more symmetric funnel plot(**Figure 10B**), indicating that the included studies had insignificant publication bias. Other outcome indicators were difficult to plot funnel plots due to insufficient number of studies to test for efficacy.

## Discussion

The prevention and treatment of DR is a global problem, and it deserves more attention in China, a country with a large diabetic population. Once NDPR progresses to the PDR stage, the damage to visual function, especially vision, will be difficult to reverse and the risk of blindness will be extremely high, so there is an urgent need for therapeutic drugs with better efficacy and higher safety to delay the development of DR. Currently, calcium hydroxybenzenesulfonate is one of the most widely used drugs for the conventional treatment of NPDR in Western medicine, and its main mechanism may be to reduce retinal albumin leakage, reduce capillary permeability, inhibit oxidative stress and aldose reductase to protect retinal tissue, improve fundus microcirculation, and control the progression of NPDR (36, 37). In recent years, retinal laser photocoagulation combined with vitreous cavity injection of anti-vascular endothelial growth factor and steroid hormone drugs has gradually become the first-line treatment option for DME (38, 39) This has also effectively controlled the progression of NPDR to PDR, but the efficacy and safety of these conventional treatment options need to be further improved. Meanwhile, in long-term clinical practice, the efficacy of TCM in preventing and treating DR has been gradually recognized, and more and more scholars have tried to explore its specific mechanism of action and find high-quality evidence-based medical evidence. There are meta-analyses proving that Chinese patent medicines such as Qiming granules (40) and Fuxiang Danshin Drops (41) and so on in combination with western medical treatment can significantly improve visual acuity and fundus signs in NPDR patients; in addition, Pang B (42) In addition, Pang B’s study included 3373 subjects with NPDR and systematically evaluated the effectiveness and safety of CHCs of the blood activating and blood stasis type in the prevention and treatment of NPDR. Compared with the conventional treatment group, placebo group and blank control group, CHCs of the blood activating and blood stasis type was effective in improving patients’ BCVA, reducing microaneurysms and lowering HbA1c index, respectively.

The types and principles of CHCs are more complex than those of proprietary Chinese medicines. The CHCs summarized in this study are all empirical formulas that have been refined by various medical practitioners in their long-term clinical practice under the guidance of TCM theory and are very effective in preventing and treating early DR. It has been shown that (43, 44) It can protect retinal microvessels by inhibiting oxidative stress in the retina, repairing damage to the blood retinal barrier, and reducing the relative expression of VEGF and up-regulating the relative expression of PEDF; Mi Meng Hua formula has the function of benefiting Qi, nourishing Yin, harmonizing Blood and brightening eyes, and previous studies have demonstrated that it can also improve the pathological state of retinal microvessels (45) It can not only inhibit the proliferation of endothelial cells under hypoxia by interfering with the intracellular VEGF-VEGFR signal transduction pathway (46) It can also reduce the excitotoxic effects of glutamate on retinal ganglion cells (47) The other CHCs in the present study were temporarily treated. Other CHCs in this study have not been reported in the literature for the relevant basic experiments.

### Effectiveness and safety of CHCs combined with conventional therapy for NPDR

The purpose of this meta-analysis was to assess the efficacy and safety of CHCs as an adjunct to the treatment of DR in terms of fundus signs, visual acuity, and other indicators. A total of 27 studies (9–35), 2144 patients met the inclusion criteria. The effectiveness results showed that the overall clinical efficiency of CHCs combined with Western medical treatment was higher compared with conventional Western medical treatment alone, including significant improvements in fundus physical indicators such as BCVA, microangioma volume and count, hemorrhagic spot area and count, and CMT, as well as laboratory tests such as glucose and lipids. Microangiomas, hemorrhagic spots, hard exudates and cotton wool spots are the main fundus signs of NPDR and are used to assess the severity of fundus lesion staging and guide clinical treatment (48, 49). In addition, macular edema is an important risk factor for exacerbating central visual impairment in NPDR (50). In addition, macular edema is an important risk factor for central visual impairment in NPDR, therefore, the assessment of macular concave thickness is important for the treatment of NPDR and the prognosis of vision (51). Hyperglycemia is the main pathogenic factor of DR, monitoring blood glucose related indicators and timely control and adjustment of blood glucose levels are essential for the prevention and treatment of DR (52). High lipid level is also a systemic risk factor that aggravates DR fundus lesions, and lipid lowering can slow down the progression of DR (53). It is necessary to test various lipoprotein indicators.

In terms of safety, a total of 8 studies (9, 17, 18, 20, 25, 29, 31, 32) provided data on adverse reactions associated with treatment. Common adverse reactions were mainly manifested as digestive symptoms (stomach upset, loss of appetite) and neurological symptoms (dizziness, hearing loss), etc. The CHCs combination treatment group experienced significantly fewer adverse events than the conventional Western medical treatment group, and the difference between the two groups was statistically significant, indicating that CHCs combined with conventional treatment for DR is safer. However, due to the lack of long-term follow-up data, long-term efficacy and safety could not be determined. Therefore, more clinical studies for long-term observation are still needed.

It’s true that there have been some studies published like this, but this study has its advantages and differences, for example, the 15 CHCs included were specific prescriptions for NPDR that recommended in the Guidelines for the Integrated Treatment of Diabetic Retinopathy, At present, there is no systematic evaluation on the efficacy and safety of the above 15 CHCs in the treatment of NPDR, it is help to strengthen the credibility of the Guidelines’ conclusions and to popularize the clinical application of CHCs mentioned above. 

### Potential limitations of the study

There are inevitable limitations of this study, firstly, the 27 included RCTs (9–35)provided insufficient completeness of the clinical trial protocols, and the transparency and standardization of the trial data were lacking; in addition, only 9 studies (9, 10, 13, 19, 21, 24, 29, 32, 34) specified the randomization method as randomized numerical table method, which may lead to selection bias of the trial data results; secondly, most of the studies did not mention how allocation concealment or blinding was conducted, and participant withdrawal and dropout The situation is unclear, which may lead to selection bias, information bias, and publication bias; finally, although we did a comprehensive literature search, there may still be unpublished literature that was not retrieved, and the included study populations were all from mainland China, and no studies of CHCs on other races were retrieved, which can only represent some Asian population trials, so the efficacy of CHCs combined with conventional Western medical treatment on other races of NPDR is unclear.

## Conclusion

In conclusion, CHCs combined with Western medicine for NPDR significantly improved clinical efficiency, best corrected visual acuity, significantly improved fundus signs such as microangioma, hemorrhage, hard exudate, cotton wool spots and CMT, and reduced the Chinese medicine evidence score, blood glucose, blood lipids and inflammatory factors, etc. In terms of safety, the proportion of adverse reactions occurring with CHCs combined with Western medicine conventional treatment was significantly lower. Therefore, this Meta-analysis proved that CHCs combined with conventional Western medicine treatment is more efficacious and has a good safety profile for the prevention and treatment of NDPR. However, the correctness of the findings of this study needs to be further confirmed, and further multicenter, large-sample, low-bias clinical studies should be conducted to find a more reliable and relevant evidence-based basis.

## Data availability statement

The original contributions presented in the study are included in the article/supplementary material. Further inquiries can be directed to the corresponding author.

## Author contributions

XL designed the study and drafted the manuscript as the first author. RH, JZ, and XS carried out the literature search. RH, XS, and ZL contributed to data extraction and quality assessment. XX and RH provided statistical supports for meta-analysis. XX supervised the study and as the corresponding author. All authors contributed to the article and approved the submitted version.

## Funding

This work was supported by National Natural Science Foundation of China(NO:81473735). The funders had no role in the study design, data collection, data analysis, interpretation, or writing of the report.

## Acknowledgments

We would like to acknowledge all the authors of the research articles used for the analysis.

## Conflict of interest

The authors declare that the research was conducted in the absence of any commercial or financial relationships that could be construed as a potential conflict of interest.

## Publisher’s note

All claims expressed in this article are solely those of the authors and do not necessarily represent those of their affiliated organizations, or those of the publisher, the editors and the reviewers. Any product that may be evaluated in this article, or claim that may be made by its manufacturer, is not guaranteed or endorsed by the publisher.
